# Antimalarial and Cytotoxic Phenolic Compounds from *Cratoxylum maingayi* and *Cratoxylum cochinchinense*

**DOI:** 10.3390/molecules14041389

**Published:** 2009-03-30

**Authors:** Surat Laphookhieo, Wisanu Maneerat, Sorwaporn Koysomboon

**Affiliations:** 1 School of Science, Mae Fah Luang University; Tasud, Muang, Chiang Rai 57100, Thailand; E-mail: wisanu_npd@hotmail.com (W.M.); 2 Faculty of Science and Industrial Technology, Prince of Songkla University, Muang, Suratthani; E-mail: sorwaporn@yahoo.com (S.K.)

**Keywords:** *Cratoxylum maingayi*, *C. cochinchinense*, Xanthones, Anti-malarial activity, Cytotoxic activity

## Abstract

Nine phenolic compounds isolated from *Cratoxylum maingayi* and *C. cochinchinense* were evaluated for anti-malarial activity against *Plasmodium falciparum*, and for cytotoxic activity against the NCI-H187 (human small cell lung cancer) cancer cell line. Formoxanthone C (**3**) was found to be the most active against the NCI-H187 cancer cell line, with an IC_50_ of 0.22 μg/mL, while vismione B (**7**) had the highest activity against *Plasmodium falciparum*, with an IC_50_ of 0.66 μg/mL.

## 1. Introduction

*Cratoxylum* is a small genus in the Clusiaceae family, which is distributed in Southeast Asian countries. Some species of this genus have been used in folk medicine by local Thai people. For example, use of the decoction of roots and stems of *C. cochinchinense* as a diuretic has been described [1]. Several xanthones have been identified from this genus [1,2,3,4,5,6,7,8,9,10] and some of these compounds have been reported to possess significant pharmacological properties, including antimalaria [2], cytotoxicity [2,3,4,5,7,10], antibacterial [3,4], and anti-HIV activity [8]. As part of our search for bioactive metabolites from Thai medicinal plants, we have evaluated the anti-malarial and cytotoxic activities of phenolic metabolites isolated from *C. maingayi* and *C. cochinchinense*. The results of our study are presented here.

## 2. Results and Discussion

**Figure 1:**
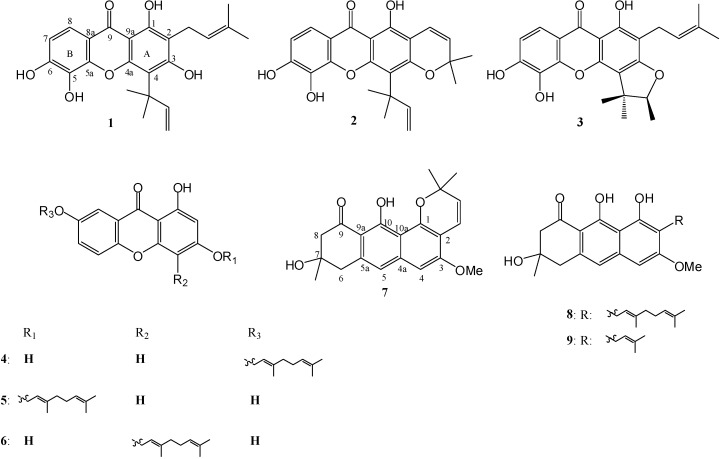
Compounds isolated from *C. maingayi* and *C. cochinchinense*.

Nine phenolic compounds (**1-9**, Figure 1) were isolated from *C. maingayi* and *C. cochinchinense*. Three of them (**1-3**) were isolated from the stem bark of *C. maingayi* and identified as 1,3,5,6-oxygenated xanthones. The remaining compounds (**4-9**) were isolated from the fruits of *C. cochinchinense* and classified into two groups: 1,3,7-oxygenated xanthones (**4-6**) and vismione derivatives (**7-9**). The compounds were characterized as gerontoxanthone I (**1**) [3], macluraxanthone (**2**) [3] and formoxanthone C (**3**) [3], 7-geranyloxy-1,3-dihydroxyxanthone (**4**) [9], cochinchinone G (**5**) [10], fuscaxanthone E (**6**) [11], vismione B (**7**) [12], vismione F (**8**) [13], and vismione E (**9**) [12] by ^1^H-NMR spectral analysis (see Experimental section) and comparison of their spectral data with values reported in the literature. Compounds **1-3** and **6** were reported for the first time as metabolites of *C. maingayi* and *C. cochinchinense*, respectively.

As summarized in Table 1, all compounds were evaluated for their anti-malarial activity against *P. falciparum* and cytotoxic activity against an NCI-H187 cancer cell line. Compounds **1**-**3**, **8,** and **9** showed strong inhibitory effect against the NCI-H187 cancer cell line and compound **3** had the highest activity, with an IC_50_ of 0.22 μg/mL whereas compounds **4**-**6** were found to be weakly active (Table 1). The anticancer drug used as a standard in our cytotoxic assay is elliticine which has an IC_50_ 0.45 μg/mL (1826.95 nM). In the case of antimalarial activity, only compounds **4**-**6** were found to be inactive, and the remaining compounds all exhibited strong inhibitory effects against *P. falciparum*, with IC_50_ values ranging from 0.66 to 3.91 μg/mL; compound **7** showed the highest activity (IC_50_ 0.66 μg/mL); the anti-malarial drug used as reference was dihydroartemisinin (IC_50_ value is 0.0011372 μg/mL, 4.0 nM).

**Table 1:** Biological activity of compounds **1**-**9**.

Compound	Anti-malarial activity^a^ IC_50_		Cytotoxicity^b^ IC_50_	
	*μ*g/mL	nM	*μ*g/mL	nM
Gerontoxanthone I ( **1**)	1.68	4,237.78	6.63	16,724.13
Macluraxanthone ( **2**)	1.35	3,422.77	3.42	8,671.02
Formoxanthone C ( **3**)	1.19	3,001.76	0.22	554.94
7-Geranyloxy-1,3-dihydroxyxanthone ( **4**)	Inactive	Inactive	10.89	28,625.22
Cochinchinone G ( **5**)	Inactive	Inactive	12.26	32,226.37
Fuscaxanthone E ( **6**)	3.02	7,938.30	20.61	54,175.01
Vismione B ( **7**)	0.66	1,862.32	1.19	3,357.82
Vismione F ( **8**)	2.02	4,758.21	6.62	15,593.74
Vismione E ( **9**)	3.91	10,970.44	not tested	not tested

^a^ Against Plasmodium falciparum; ^b^ Against human lung cancer (NCI-H187)

It should be noted that the only structural difference between xanthone **1** and **3** is at C-3 and C-4. Compound **1** possesses a hydroxyl and isoprenyl groups at C-1 and C-2, respectively, while **3** has a α,α,β-trimethylfuran ring on C-3/C-4, which plays an important role in the cytotoxicity. Compounds **1** and **2**, which contain a 1,1-dimethyl-2-propenyl moiety at C-4, seemed to have reduced cytotoxicity. For anti-malarial activity, compounds **1**-**3** exhibited strong activity against *P*. *falciparum*, therefore, substituent groups on ring A had no effect on this activity. The side chain of compounds **4**-**6** had a slightly effect on cytotoxicity, but the geranyl side chain of **6** seemed to be much more effective in terms of anti-malarial activity. Structural variation between compounds **7** and **9** also results in remarkably different activity. Again the only structural differences between **7** and **9** are the substituents at C-1 and C-2. Compound **7** contains a chromene ring at C1/C-2, but **9** has a hydroxyl and isoprenyl groups at C-1 and C-2, respectively. This chromene ring appears to be particularly responsible for the cytotoxic and anti-malarial activity.

## 3. Experimental

### 3.1. General

The ^1^H- and ^13^C-NMR spectra were recorded using 300 MHz Bruker spectrometer. Chemical shifts were recorded in parts per million (δ) in CDCl_3_ (unless otherwise specified) with tetramethylsilane (TMS) as the internal reference. Quick column chromatography (QCC) and column chromatography (CC) were carried out on silica gel 60 H (Merck, 5-40 μm) and silica gel 100 (Merck, 63-200 μm), respectively. Precoated plates of silica gel 60 F_254_ were used for the analytical TLC.

### 3.2. Plant material

Stem bark of *C. maingayi* was collected from Nong Khai Province, in the northeastern part of Thailand in December 2007, while the fruits of *C. cochinchinense* were collected from Mae Fah Luang University, Tasud, Muang, Chiang Rai Province, in the northern part of Thailand in December 2006. Botanical identifications were achieved by comparison with a voucher specimen No. SL-5 (PSU) for *C. maingayi* and SL-1 (PSU) for *C. cochinchinense* held in the herbarium collection of the Department of Biology, Prince of Songkla University, Songkhla, Thailand.

### 3.3. Extraction and isolation

The stem bark of *C. maingayi* (4 kg) was extracted with hexane (15 L) and EtOAc (15 L), respectively, over a period of three days each at room temperature and the extracts were evaporated under reduced pressure to provide crude hexane (25 g) and EtOAc extracts (40 g), which were combined (65 g) and subjected to QCC eluting with a gradient of hexane-EtOAc (100% hexane to 100% EtOAc) to afford 15 fractions (CMB1-CMB15). Fraction CMB8 (570 mg) was purified by CC using 25% EtOAc-hexane to afford compound **1** (20 mg). Fraction CMB10 (2.20 g) was repeatedly subjected to CC using 40% EtOAc-hexane to give 5 subtractions (CMB10A-E). Compounds **2** (5 mg) and **3** (8.7 mg) were derived from subfraction CMB10D (42 mg) by prep. TLC using 2% MeOH-DCMas eluent. Isolation and purification of compounds **4** and **6**-**9** from the hexane and EtOAc extracts of dried fruits of *C. cochinchinense* have been reported earlier [14]. Compound **5** (13.5 mg) was also isolated from the EtOAc extract by repeated CC with 100% DCM of fraction E9a (352.7 mg).

*Gerontoxanthone I* (**1**): ^1^H-NMR: δ 13.62 (1H, s, 1-OH), 7.72 (1H, d, *J* = 9.0 Hz, H-8), 6.94 (1H, d, *J* = 9.0 Hz, H-7), 6.68 (1H, dd, *J* = 17.7, 10.5 Hz, H-2′′), 5.29 (1H, d, *J* = 17.7 Hz, H-3a′′), 5.24 (1H, br t, *J* = 6.9 Hz, H-2′), 5.13 (1H, d, *J* = 10.5 Hz, H-3b′), 3.47 (1H, d, *J* = 6.9 Hz, H-1′), 1.86 (3H, s, H-4′), 1.79 (3H, s, H-5′), 1.68 (6H, s, H-4′′ and H-5′′).

*Macluraxanthone* (**2**): ^1^H-NMR: δ 13.52 (1H, s, 1-OH), 7.68 (1H, d, *J* = 9.0 Hz, H-8), 6.93 (1H, d, *J* = 9.0 Hz, H-7), 6.78 (1H, d, *J* = 9.9 Hz, H-2′), 6.75 (1H, dd, *J* = 17.7, 10.5 Hz, H-2′′), 5.60 (1H, d, *J* = 9.9 Hz, H-1′), 5.22 (1H, d, *J* = 17.7 Hz, H-3a′′), 5.04 (1H, d, *J* = 10.5 Hz, H-3b′′), 1.64 (6H, s, H-4′′, H-5′′), 1.51 (6H, s, H-4′, H-5′).

*Formoxanthone C* (**3**): ^1^H-NMR: δ 13.40 (1H, s, 1-OH), 7.73 (1H, d, *J* = 9.0 Hz, H-8), 6.92 (1H, d, *J* = 9.0 Hz, H-7), 5.28 (2H, t, *J* = 6.9 Hz, H-2′), 4.52 (1H, q, *J* = 6.6 Hz, H-2′′), 3.30 (2H, d, *J* = 6.9 Hz, H-1′), 1.78 (3H, s, H-5′), 1.69 (3H, s, H-4′), 1.57 (3H, s, H-5′′), 1.43 (3H, d, *J* = 6.6 Hz, H-3′′), 1.31 (3H, s, H-4′′).

*7-Geranyloxy-1,3-dihydroxyxanthone* (**4**): ^1^H-NMR (CDCl_3_+MeOD): δ 12.98 (1H, s, 1-OH), 7.60 (1H, d, *J* = 2.4 Hz, H-8), 7.33 (1H, d, *J* = 9.1, H-5), 7.28 (1H, dd, *J* = 9.1, 2.4, H-6), 6.42 (1H, d, *J* = 1.8, H-4), 6.36 (1H, d, *J* = 1.8, H-2), 5.51 (1H, br t, *J* = 6.6, H-2′), 5.10 (1H, br t, *J* = 6.6, H-6′), 4.63 (2H, d, *J* = 6.6, H-1′), 2.12 (4H, m, H-4′, H-5′), 1.78 (3H, s, H-8′), 1.67 (3H, br s, H-10′), 1.61 (3H, br s, H-9′).

*Cochinchinone G* (**5**): ^1^H-NMR: δ 12.70 (1H, s, 1-OH), 7.58 (1H, d, *J* = 3.0 Hz, H-8), 7.29 (1H, d, *J* = 9.0 Hz, H-5), 7.24 (1H, dd, *J* = 9.0, 3.0 Hz, H-6), 6.39 (1H, d, *J* = 3.0 Hz, H-4), 6.33 (1H, d, *J* = 3.0 Hz, H-2), 5.48 (1H, t, *J* = 6.6 Hz, H-2′), 5.09 (1H, t, *J* = 6.6 Hz, H-6′), 4.62 (2H, d, *J* = 6.6 Hz, H-1′), 2.12 (2H, m, H-5′), 2.11 (2H, m, H-4′), 1.76 (3H, s, H-9′), 1.67 (3H, s, H-10′), 1.55 (3H, s, H-8′).

*Fuscaxanthone E* (**6**): ^1^H-NMR (CDCl_3_+MeOD): δ 12.80 (1H, s, 1-OH), 7.49 (1H, d, *J* = 2.7 Hz, H-8), 7.31 (1H, d, *J* = 9.0 Hz, H-5), 7.24 (1H, dd, *J* = 9.0, 2.7 Hz, H-6), 6.42 (1H, d, *J* = 2.1 Hz, H-4), 6.33 (1H, d, *J* = 2.1 Hz, H-2), 5.48 (1H, t, *J* = 6.6 Hz, H-2′), 5.08 (1H, t, *J* = 6.6 Hz, H-6′), 4.62 (2H, d, *J* = 6.6 Hz, H-1′), 2.12 (4H, m, H-4′, H-5′), 1.76 (3H, s, H-9′), 1.67 (3H, s, H-10′), 1.55 (3H, s, H-8′).

*Vismione B* (**7**): ^1^H-NMR: δ 14.69 (1H, s, 10-OH), 6.71 (1H, s, H-4), 6.62 (1H, d, *J* = 9.9 Hz, H-1′), 6.40 (1H, s, H-5), 5.52 (1H, d, *J* = 9.9 Hz, H-2′), 3.84 (3H, s, 3-OMe), 2.96 (2H, s, H-6), 2.74 (2H, s, H-8), 1.48 (3H, s, H-4′), 1.45 (3H, s, H-5′), 1.34 (3H, s, H-1′′).

*Vismione F* (**8**): ^1^H-NMR: δ 16.12 (1H, s, 10-OH), δ 9.44 (1H, s, 1-OH), 6.84 (1H, s, H-4), 6.52 (1H, s, H-5), 5.23 (1H, m, H-2′), 5.06 (1H, m, H-6′), 3.90 (3H, s, 3-OMe), 3.44 (2H, d, *J* = 6.9 Hz, H-1′), 2.96 (2H, s, H-6), 2.74 (2H, s, H-8) 1.93-2.06 (4H, m, H-4′ and H-5′), 1.63 (6H, s, H-8′ and H-10′), 1.56 (3H, s, H-9′), 1.43 (3H, s, H-1′′).

*Vismione E* (**9**): ^1^H-NMR: δ 16.14 (1H, s, 10-OH), 9.95 (1H, s, 1-OH), 6.85 (1H, s, H-4), 6.53 (1H, s, H-5), 5.23 (1H, t, *J* = 6.9 Hz, H-2′), 3.92 (3H, s, 3-OMe), 3.43 (2H, d, *J* = 6.9 Hz, H-1′), 3.04 (2H, s, H-6), 2.83 (2H, s, H-8), 1.80 (3H, s, H-4′), 1.68 (3H, s, H-5′), 1.43 (3H, s, H-1′′).

### 3.4. Anti-malarial assay

Antimalarial activity was evaluated against the parasite *P. falciparum* (K_1_, multidrug resistant), using the method of Trager and Jensen [15]. Quantitative assessment of *in vitro*malarial activity was determined by means of the microculture radioisotope technique based on the method described by Desjardins *et al*. [16] The inhibitory concentration (IC_50_) represented the concentration that caused 50% reduction in parasite growth which was indicated by the *in vitro* uptake of [^3^H]-hypoxanthine by *P. falciparum*. The standard compound was dihydroartemisinin (IC_50_ 0.0011372 μg/mL; 4.0 nM).

### 3.5. Cytotoxic assay

The procedure for cytotoxic assay was performed by a colorimetric method (anti-NCI-H187) as described by Skehan *et al*. [17]. Ellipticine was the reference substance in this study and the IC_50_ value is 0.45 μg/mL (1826.95 nM) for anti- NCI-H187.

## 4. Conclusions

We have reported the *in vitro* anti-malarial and cytotoxic activity of nine phenolic compounds isolated from *C. maingayi* and *C. cochinchinense* in Thailand. Most of the phenolic compounds displayed significant inhibition to the growth of *P. falciparum* and a human cancer cell line (NCI-H187), *in vitro*. Among the phenolic compounds tested, xanthone **3** (a 1,3,5,6-oxygenated xanthone) bearing a α,α,β-trimethylfuran ring on C-3/C-4, showed the most potent cytotoxic activity against NCI-H187 (IC_50_, 0.22 μg/mL), which is stronger than that of elliptecine, a standard drug (IC_50_, 0.45 μg/mL). In addition, all biological activities of 1,3,5,6-oxygenated xanthones **1**-**3** was stronger than that of 1,3,7-oxygenated xanthones (**4**-**6**) probably due to the presence of two hydroxyl groups at C-5 and C-6 in xanthones **1**-**3** which are important for enhancing the cytotoxic activity cytotoxicity against NCI-H187 cancer cell line and anti-malarial activity.
